# Immediate effects of water resistance therapy on patients with vocal fold mass lesions

**DOI:** 10.1007/s00405-020-05887-y

**Published:** 2020-03-14

**Authors:** Matthias Echternach, Julius Raschka, Liudmila Kuranova, Marie Köberlein, Bernhard Richter, Michael Döllinger, Marie-Anne Kainz

**Affiliations:** 1 Division of Phoniatrics and Pediatric Audiology, Department of Otorhinolaryngology, Munich University Hospital (LMU), Marchioninistr. 15, 81377 Munich, Germany; 2 Freiburg Institute of Musicians’ Medicine, and Medical Faculty, Freiburg University, Elsässerstr. 2, 79114 Freiburg, Germany; 3 Division of Phoniatrics and Pediatric Audiology at the Department of Otorhinolaryngology Head and Neck Surgery, University Hospital Erlangen, FAU Erlangen-Nürnberg, Waldstrasse 1, 91054 Erlangen, Germany

**Keywords:** Vocal fold mass lesion, Water resistance therapy, High-speed imaging, EGG

## Abstract

**Introduction:**

Semi-occluded vocal tract exercises, such as water resistance therapy (WRT), are widely used in voice therapy. However, the potential positive effects of such a therapy on vocal fold oscillation patterns in patients indicating a need for phonomicrosurgery have not yet been explored. The presented study aims to analyze the effect of WRT in patients suffering from vocal fold mass lesions.

**Materials and methods:**

Eight participants with vocal fold mass lesions were asked to sustain a phonation on the vowel /i/ at a comfortable loudness and a fundamental frequency of 250 Hz (females) or 125 Hz (males). During phonation the subjects were simultaneously recorded with transnasal high-speed videoendoscopy (HSV, 20.000 fps), electroglottography, and audio signals. These subjects then performed a WRT (phonation in a silicone tube of 30 cm length, 5 cm below the water surface) for 10 min. Repeated measurements of sustained phonation were performed 0, 10, and 30 min after exercising. From the HSV data the glottal area waveform (GAW) was segmented and GAW parameters were computed.

**Results:**

During WRT there was an increase of the GAW related open quotient and closing quotient. Immediately after WRT, there was a drop of both values followed by a rise of these parameters up to 30 min after the intervention. Furthermore, there was no correlation between GAW and electroglottographical open quotients.

**Conclusions:**

The effects observed after a single session of WRT on participants with vocal fold mass lesions showed a similar pattern to vocal fatigue.

## Introduction

Vocal fold mass lesions are a frequent cause for dysphonia. The occurrence of numerous entities, including polyps, Reinke edemas, and nodes, on the oscillating vocal folds could contribute to impairment of voice source production [1]. The effect of vocal fold mass lesions on voice production is not homogenous. In this respect, some mass lesions might change stiffness within the vocal fold, whereas others might increase the mass itself. In both cases, the oscillation patterns might be influenced, with the likelihood of aperiodicities increasing [2]. The oscillation pattern and/or frequency might also differ between the left and right vocal fold with the consequence of asymmetries or left–right phase. It has been shown that vocal fold asymmetries reduce the strength of intraglottal vortices and thus vocal efficiency [3]. Furthermore, some mass lesions, like polyps, might also prevent the closure of the entire membranous part of the vocal folds. As a consequence, there could be a lack of interruption of the transglottic airflow by the oscillating vocal folds, inducing vortices and decreasing signal to noise ratio [4]. However, not all mass lesions necessarily influence voice production. In some cases, vocal fold oscillation patterns are not influenced by vocal fold mass lesions and vocal fold closure is possible along the entire membranous part of the vocal folds [5, 6]. Therefore, the most suitable course of therapy should be decided with a focus on the vocal function and vocal demands of the patient rather than solely on the verification of the mass lesion.

Different approaches are appropriate in specific cases where a need for medical therapy is identified. While some acute edemas could be treated by pharmacotherapy, i.e., corticosteroids, other voice problems could be treated using voice therapy or phonomicrosurgery [1]. To the best of the authors’ knowledge there are currently no studies available analyzing the special cases in which a voice therapy by voice and speech pathologists could be considered advantageous compared to phonomicrosurgery. Also, there are no orienting tests available which could predict the success of non-surgical therapy.

For dysphonic patients there are many approaches to non-surgical voice therapies. During recent decades, specific scientific interest has been developed in semi-occluded vocal tract exercises (SOVT). In general, such exercises are characterized by a semi-occlusion at the tongue or lips, or extending the vocal tract by tube systems. In this respect, there is a difference between SOVT exercises that use tubes ending in free air [7] or under water [8, 9]. The latter are referred to as water resistance therapies (WRT) where LaxVox© and DoctorVox© are the commercially available products [10]. For WRT, the depth to which the tube is submerged under water defines the intraoral pressure and therefore influences the transglottic pressure difference [11]. Furthermore, the popping bubbles produced in the water cause a fluctuating intraoral pressure during phonation. It is thought that such pressure changes might have a massage effect within the vocal tract [11].

WRT has multiple effects on voice production: during the exercise the vertical laryngeal position is lowered [12], the intraoral rise of pressure leads to an increase of the subglottic pressure [13], fundamental frequency is often changed [13–15], the phonation collision threshold pressure after WRT is increased, the closed quotient is diminished [11], and the vocal quality is improved [16, 17].

In general, WRT is performed many times a day with the expectation that the body memory of voice production is positively influenced and thus a more efficient voice is produced. If performed for a single performance, WRT is expected to only have a short-term effect on voice production. In a recent study, it has been shown that for non-dysphonic voices, WRT with a tube of 30 cm, inner diameter 9 mm, submerged 5 cm under water only had an effect on lower frequency perturbation for 5 min [18]. The effect of such a WRT on vocal fold oscillation pattern in patients with vocal fold mass lesions has not yet been clarified.

The presented study aims to analyze the effect of a defined short-term water resistance therapy in participants suffering from organic-based dysphonia, i.e., a vocal fold mass lesion where the need for phonomicrosurgery was indicated.

## Materials and methods

### Subjects

**Fig. 1:**
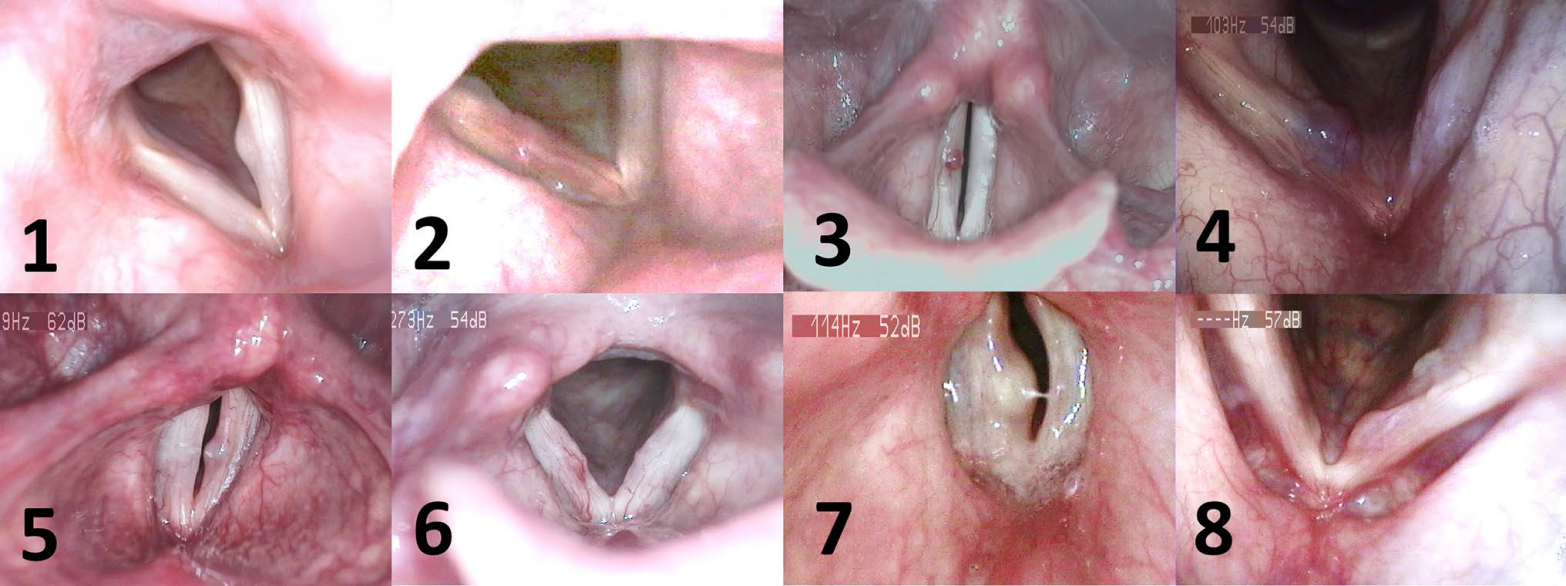
Laryngoscopic images of the different vocal fold mass lesions for each subject

After approval from the local ethical committee, eight participants were included in the study. Multidimensional voice evaluation was conducted by means of the protocol of the European Laryngological Society [19]. Two experienced laryngologists/phoniatricians with professional experience for more than 15 years agreed conservative therapy (i.e., voice therapy and/or pharmacotherapy) as inappropriate, and phonomicrosurgery was recommended for all the participants. Table 1 shows age, gender, pathology, Voice Handicap Index (VHI) [20, 21], and the Dysphonia Severity Index (DSI) [22] and Fig. 1 shows laryngoscopical pictures for each subject.

**Table 1:** Gender, age, pathology, lateralization, Dysphonia Severity Index (DSI), Voice Handicap Index (VHI), and fundamental frequency (*f*_o_) range

Subject	Gender	Age	Pathology	Lateralization	DSI	VHI	*f*_o_ range
1	f	50	Cyst	Left	0.6	47	143–573
2	f	51	Edema	Right	2.1	36	99–310
3	f	60	Polyp	Right	3.5	12	143–380
4	f	43	Polyp	Right	1.2	56	112–441
5	m	28	Cyst	Left	4.5	23	102–551
6	f	43	Cyst	Right	2.4	48	124–466
7	m	38	Cyst	Right	5.4	33	80–787
8	f	59	Polyp	Left	− 1.2	66	176–405

### Task and intervention

The subjects were asked to sustain phonation on the vowel /i/ at a comfortable loudness, with a fundamental frequency (*f*_o_) of 250 Hz for the female and 125 Hz for the male voices. These *f*_o_s were chosen because these were assumed being in the gender related *f*_o_ speaking region. During phonation, the subjects were simultaneously recorded with transnasal high-speed videoendoscopy (HSV), electroglottography, and microphone. After the initial recording (pre) the subjects performed a WRT for 10 min with a 30 cm tube, diameter 9 mm, submerged 5 cm under the surface of the water. The depth of 5 cm was chosen because it was assumed that the effect on vocal fold oscillations would be greater compared to lower values of depth. However, not as great so that strong compensations might be observed, as shown before [11]. To keep the depth of the tube under water constant, the 5 cm depth was marked at the tube. Immediately after insertion of the tube into the water and stabilization of phonation during WRT, the WRT recording was performed at almost stable pitch and loudness. During WRT and after the WRT recording, the subjects were asked to change their loudness at comfortable pitch and vary pitch at comfortable loudness to vary tension of the vocal folds and thus induce a stretching effect during WRT. Directly after WRT (0) as well as 10 and 30 min after WRT, recordings of sustained phonation were repeated.

### Recordings

High-speed videoendoscopy (HSV, Fastcam SA-X2, Photron, Tokyo, Japan) was performed, similar to previous studies [6, 23, 24], using transnasal endoscopy with a flexible endoscope (ENF GP, Fa. Olympus, Hamburg, Germany) with a frame rate of 20.000 fps and a spatial resolution of 386 × 320 pixels. Simultaneously, audio signals (IMK SC 4061 microphone, DPA microphones, Alleroed, Denmark or Sennheiser ME 62, Sennheiser, Wedemark, Germany) and electroglottographic (EGG) signals (EG2-PCX2, Glottal Enterprises, Syracuse, NY) were recorded. During the experiment, no anesthetic medication was applied for the transnasal endoscopy. The audio signal was calibrated with a sound level meter (Voltcraft, Hong Kong, China) using the Sopran software (Svante Granqvist, Karolinska, Stockholm, Sweden). The HSV videos were post-processed by means of rotation, fast Fourier treatment, and cropping as described in [25]. The calculation of the glottal area waveform (GAW) and phonovibrograms from the HSV images was performed as described previously [26, 27].

### Analysis

For comparison, 300 ms time windows were constructed. To exclude the voice onset, these windows were started approximately 5 s after voice onset. For these windows, mean values for glottal area-derived open quotient (OQ_GAW_), electroglottographical open quotient (OQ_EGG_), sound pressure level (SPL), closing quotient (ClQ, closing phase/period), Jitter, and fundamental frequency (*f*_o_) were calculated using the multi-signal analyzer (Schäfer/Schlegel, FAU Erlangen-Nürnberg, Germany) (Table 2).

**Table 2:** Measures and origin

Glottal area waveform	Electroglottography	Audio
Closing quotient (ClQ)	–	–
Open quotient(OQ)	Open quotient (OQ)	–
–	Fundamental frequency (*f*_o_)	Sound pressure level (SPL)
Jitter (first)	Jitter (first)	Jitter (first)

Due to the process of segmentation, a tolerance threshold of 5% from the base value of the GAW amplitude was set for detection of OQ_GAW_, thus the glottis was presumed closed for the lower values. OQ_EGG_ was calculated according to the Howard criterion [28], thus defining the contact by the positive peak of the derivative EGG signal and the decontacting by the crossing of 4/7 of the amplitude. For frequency perturbation, the Jitter (first) for all three voice signals (GAW, EGG, and audio) was measured.

Pearson correlation was used and due to the small sample size (*n* = 8) comparative statistics was considered not meaningful.

## Results

**Fig. 2:**
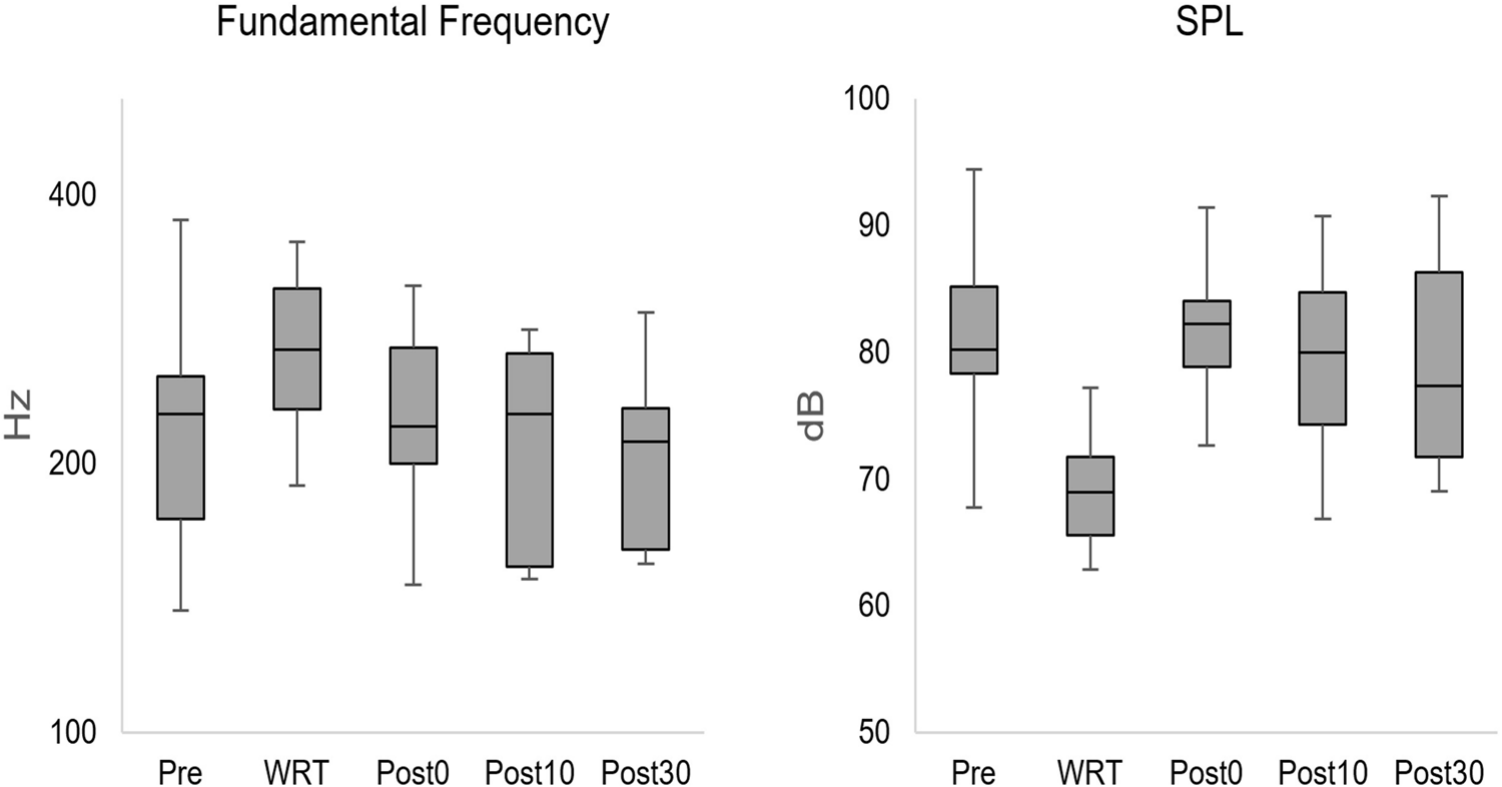
Boxplots concerning fundamental frequency and sound pressure level (SPL) with respect to the points of measurement [pre = pre intervention, water resistant therapy (WRT), post 0, 10, and 30 relate to the minutes after the intervention]

All subjects were able to fulfill the task without any interruption. During the WRT recording at the beginning of the WRT, *f*_o_ was increased and returned almost to the initial value immediately afterwards (Fig. 2).

**Fig. 3:**
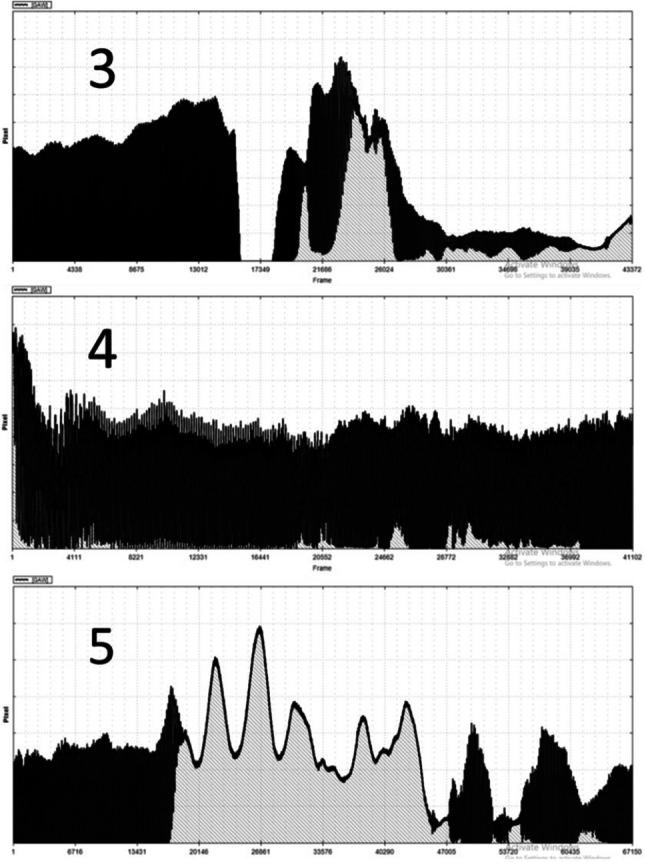
Glottal area waveform for subjects 3, 4, and 5 for the time of insertion of the tube into water. Black refers to the GAW amplitude whereas the grey under the GAW refers to an open glottis

There were strong inter-individual differences concerning how the transition from the /i/ vowel to WRT took place. As can be seen in Fig. 3, during the insertion of the tube into the water, subject 3 exhibited a strong adduction between the epiglottis and the arytenoid cartilages and consequently the GAW equaled zero. The effects of pressure releases due to the bubbles then became visible in the GAW. Subject 4 had a rather smooth transition during the insertion of the tube into water and performed phonation throughout the WRT task. For subject 5 the insertion had the consequence of an aphonic phase, where only the bubbles but no vocal fold oscillations were visible. Later, the subject started phonation again with visible changes of amplitudes due to the bubble related pressure differences.

**Fig. 4:**
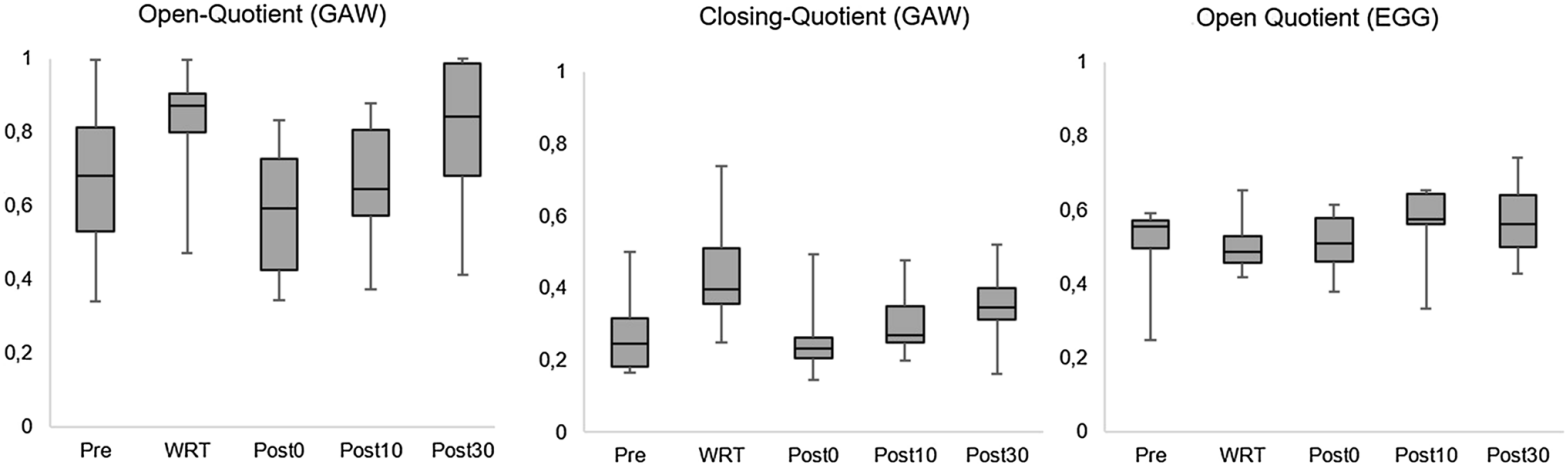
Boxplots concerning open quotient and closing quotient for the glottal area waveform and open quotient for the electroglottographical signal (EGG) with respect to the points of measurement [pre = pre intervention, water resistant therapy (WRT), post 0, 10, and 30 relate to the minutes after the intervention]

**Fig. 5:**
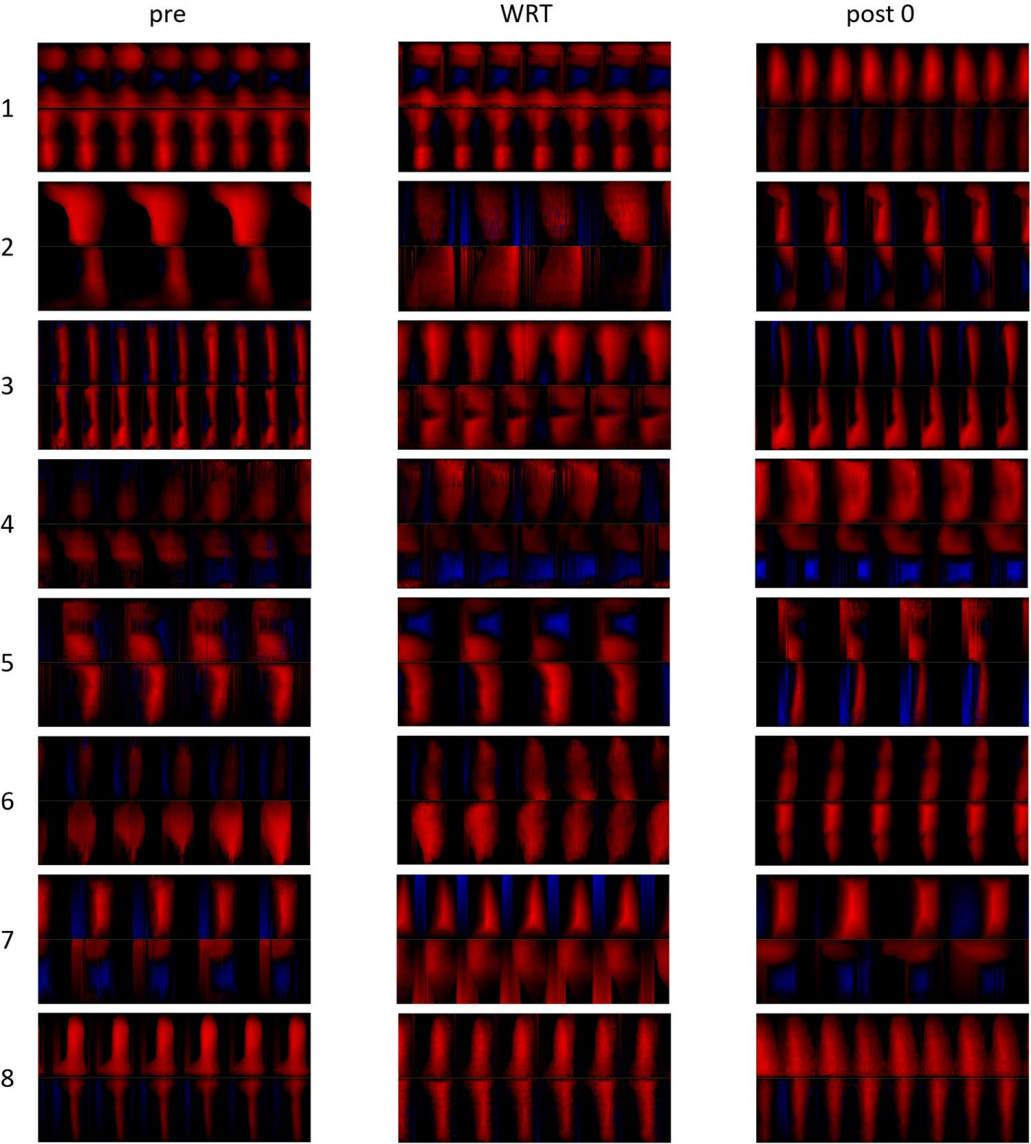
Phonovibrograms of all subjects performing a vowel /i/ before, during, and immediately after WRT. The PVGs refer to a 25 ms window at the middle of the analyzed 300 ms window

During WRT there were strong changes of vocal fold oscillation patterns. As can be seen in Fig. 4, both the OQ_GAW_ and ClQ were risen. In contrast, there was no such increase of OQ_EGG_. The Pearson correlation between OQ_GAW_ and OQ_EGG_ was *r* = 0.002, *p* > 0.05. As can be seen in the phonovibrograms (Fig. 5), there was no obvious different behavior concerning the lateralization, i.e., the vocal fold exhibiting the mass lesion showed a different behavior during WRT than the other.

**Fig. 6:**
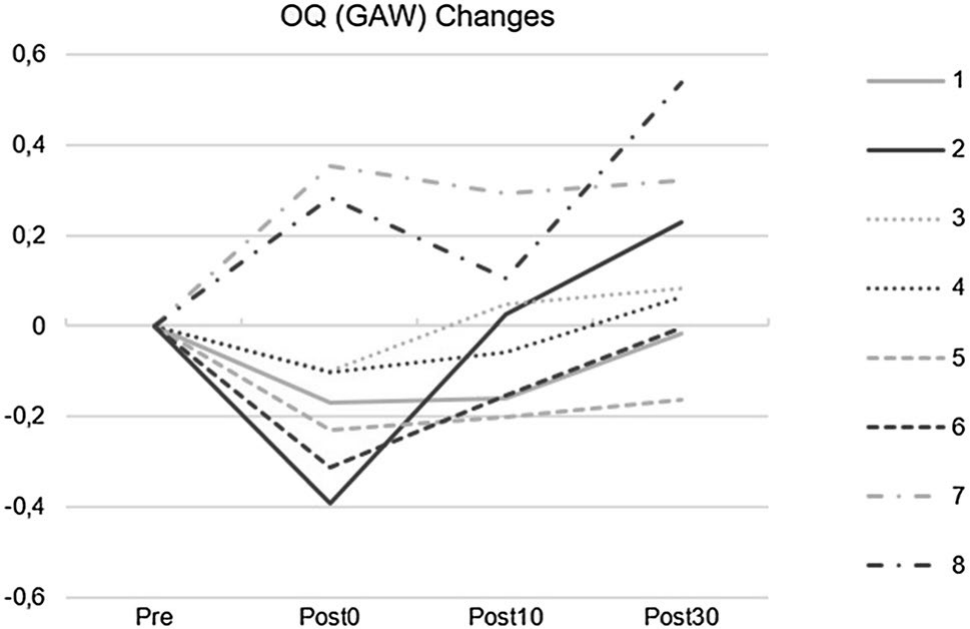
Changes of the glottal area waveform (GAW) for all subjects relative to the pre value

Immediately after WRT, *f*_o_ showed a decrease followed by a further minor decrease 30 min after WRT, although *f*_o_ was provided in front of every measurement. OQ_GAW_ showed immediately after WRT, a lower value compared to the pre-measurement, but up to 30 min after WRT it rose to a greater value in comparison to the pre-measurement. A comparable rise was also found for ClQ. However, there were large inter-individual differences concerning the course of all measurements: as can be seen in Fig. 6, where six of the subjects showed lower values directly after WRT, while there were two subjects with greater values.

**Fig. 7:**
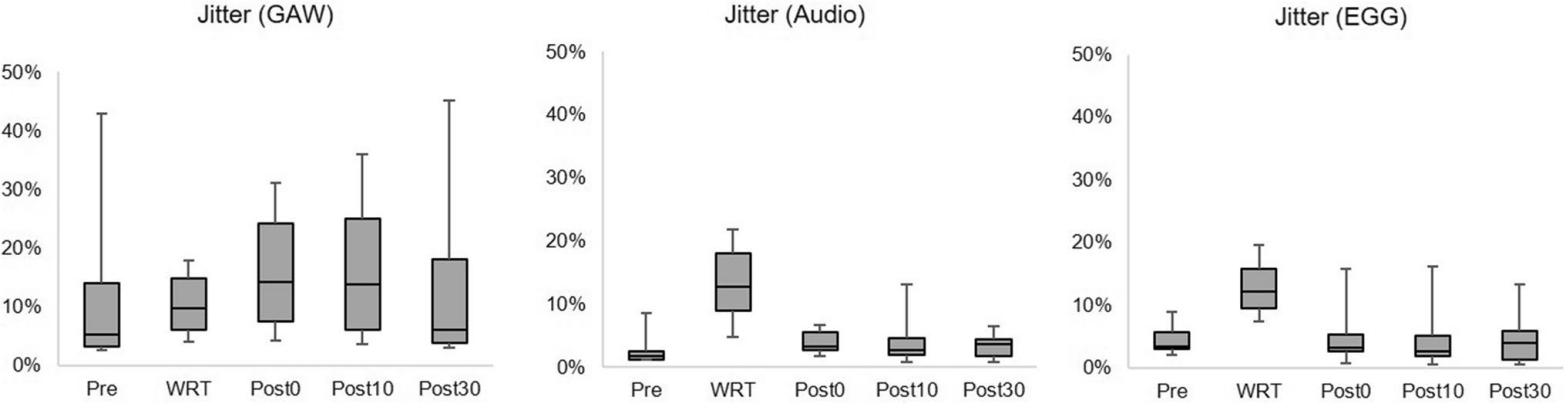
Boxplots concerning Jitter for the electroglottographical (EGG), the audio, and glottal area waveform (GAW) signal with respect to the points of measurement [pre = pre intervention, water resistant therapy (WRT), post 0, 10, and 30 relate to the minutes after the intervention]

During WRT there was an increase of different Jitter measurements for all signals (Audio, EGG, GAW) (Fig. 7). However, except for Jitter_GAW_, which shows a slight increase directly and 10 min after WRT, the remaining values are almost unchanged after WRT.

## Discussion

This study analyzed the effects of water resistance therapy in participants with vocal fold mass lesions. In general, strong effects were found during the WRT intervention with some effects observed after WRT, which, in part, could be considered signs of vocal fatigue.

WRT is considered an important technique in voice therapy [8, 11, 29, 30]. There are some effects that are thought to be helpful for dysphonic patients. In this respect, besides effects on vocal fold oscillations [11, 30] it has been assumed that WRT produces a massage effect during the intervention [11]. For healthy voices the buildup and release of bubbles is detectable in both the electoglottographical and glottal area wave form voice signals [11, 30]. The presented data also show such an effect in both GAW and EGG signals, for participants with vocal fold mass lesions. OQ_GAW_ also rose during WRT as well as ClQ and Jitter. However, in contrast to electroglottographical studies in non-dysphonic voices [30], there was no concurrent rise of OQ_EGG_. Those OQ measures are not equal, where OQ_GAW_ describes the two-dimensional view of the glottis above, and OQ_EGG_ is related to the three-dimensional contact area between the vocal folds. For non-dysphonic voices, previous investigations found a strong correlation between OQ_GAW_ and OQ_EGG_ for OQ_GAW_ values approximately below 0.7, but a weak correlation for values above [31]. In contrast to non-dysphonic voices, in the presented study there was no correlation between OQ_GAW_ and OQ_EGG_. One explanation is that the mass lesions could lead to a—compared to healthy voices—different electrical conductivity. As mass lesions could bridge the glottal gap earlier during vocal fold closing and later during the opening, there could be an increase of the EGG amplitude at the same time when the glottis is still open anteriorly or posteriorly to the mass lesion. As a consequence, for subjects with vocal fold mass lesions the authors suggest that OQ_EGG_ values should be interpreted with caution.

Other than a rise of the open and closing phase, the phonovibrograms for most participants exhibited no great changes of the general oscillation pattern during or after WRT. As only one vocal fold had the mass lesion, it could be expected that the healthy vocal fold might react differently to the WRT than the vocal fold with the mass lesion. On one hand, the mass lesion increases the vocal fold mass itself, but on the other hand it frequently results in increased stiffness. Because the eigenmode of both vocal folds should be comparable for a homogenous oscillation pattern, muscular adjustments to achieve comparable eigenmode should differ between the left and right vocal fold. Because the rise of pressure during WRT will have a stronger effect on soft tissue, it could be assumed that the healthy vocal fold would react stronger after WRT. However, this was not the case in the presented study. It seems that WRT influences the general oscillatory system more than one side.

The data corresponding to the insertion of the tube into the water showed three different behaviors. The first behavior exhibited no great changes of GAW amplitudes during the insertion, as presented by subject 4. Supraglottic compression occurred in subject 3 while subject 5 suddenly stopped phonating. The latter two behaviors are presumably a consequence of the rise of intraoral pressure. It is unclear why some subjects tried to avoid the rise of pressure by supraglottic contraction whereas other subjects tolerated aphonia until the pressure difference between subglottic and supraglottic pressure increased and enabled phonation again. It could be hypothesized that the first behavior should be associated with very stable phonation. However, as can be seen in Fig. 6, the OQ_GAW_ changes were almost comparable for all three patterns for subjects 3, 4, and 5.

Most of the subjects exhibited a lower OQ_GAW_ immediately after WRT. This could be the result of greater efficiency perhaps caused by a stretching and massage effect of WRT. However, a noticeable increase in OQ_GAW_ was observed for two subjects. The reason for this is not clear. Interestingly, there was a great increase of OQ_GAW_ with time after WRT for most subjects, with greater values 30 min after than before the WRT for 5 out of 8 subjects. At the same time, ClQ showed greater values and SPL was slightly diminished. Thus, WRT leads to a lower vocal efficiency given that the subglottic pressure was almost unchanged. These changes could therefore be considered as a sign of vocal fatigue as negative adaptation or vocal effort. It could be speculated that the WRT might produce vocal loading too great to be tolerated by participants with vocal fold mass lesions. How much loading is produced by a WRT has not yet been analyzed, neither in healthy nor dysphonic subjects. If the results are a consequence of vocal fatigue or vocal effort, they could occur immediately, but also minutes after vocal loading. Therefore, it could be speculated that the two subjects exhibiting great OQ_GAW_ values immediately after WRT had lower tolerance to vocal loading during WRT. In this context, it could be assumed that participants exhibiting low values for the DSI and high for the VHI could have a lower tolerance. However, analyzing subjects 1, 4, and 8 which present such a constellation showed no clear tendency. Out of these subjects, only participant 8 exhibited a greater rise for OQ_GAW_. Unfortunately, although all subjects were asked to perform sustained phonation and pitch glides during WRT, control of SPL was not possible due to the tube being submerged under water. Therefore, it is possible that these subjects used a greater SPL and therefore greater loading during WRT.

In contrast to another study [11], the presented data revealed no or possibly an inverse impact of WRT regarding perturbation values after WRT. The Jitter did not decline at any measurement time after the intervention. In fact, there is an immediate increase in Jitter_GAW_, which only returns to its initial value after 30 min. Hence, it can be concluded that overall there were no positive long-term effects of WRT regarding this participant cohort after a single performance. It could be useful to study such effects of vocal fatigue in participants exhibiting vocal fold mass lesions not only with regard to WRT but using vocal loading tests, for example, a loading of 10 min reading a text greater than 80 dB(A) measured in a distance of 30 cm [32], to estimate vocal capacity in future investigations. Furthermore it would be of interest to measure the level of vocal loading produced by WRT.

There are some important limitations of this study. This study only analyzes participants for whom phonomicrosurgery was indicated as the appropriate treatment. It might be that patients with vocal fold mass lesions whose vocal function is not greatly impaired [5, 33] or with nodes which are considered to be effectively treated by voice therapy could behave differently. If so, such a WRT could be a good predictor of therapeutic success. It should be stated in this context that the presented data are not contradicting a generally positive effect of WRT. In the presented study a single performance of WRT was performed. When used in therapy, multiple WRT sessions a day are commonly recommended. Therefore, it cannot be excluded that long-term WRT might also be beneficial for patients exhibiting vocal fold mass lesions. In addition, only one water depth of 5 cm was chosen. Different depths would produce different pressures that could have varying effects on a comparable participant collective. Furthermore, the extensive dataset collected, i.e., a single measurement of 9 s for each time point produced more than 32 GB of data, restricted the number of subjects to 8, when a greater collective of participants might exhibit greater or different effects. We hope that studies including more participants will be possible in the future to present, on one hand, comparative statistics, and on the other hand, allow separation of different entities of vocal fold mass lesions. The participants were analyzed at the given *f*_o_ of 125 Hz for male and 250 Hz for female voices, respectively, which was assumed being in the *f*_o_ region of the speaking pitch. It cannot be excluded that measurement at a different *f*_o_ could provide different results. Also, the task was fixed by varying *f*_o_ for a comfortable loudness and varying loudness for a comfortable *f*_o_. The interpretation of “comfortable” might vary among subjects and therefore might influence the results. However, for untrained subjects it appears difficult to fix both loudness and *f*_o_ at the same time. Finally, the experiment was performed using a flexible transnasal endoscopy which could sometimes be associated with the gag reflex and glottal adduction. Although there was no gag reflex during the experiment it cannot be excluded that OQ_GAW_ was influenced by these circumstances. However, because endoscopy was performed throughout the experiment, this potential limitation should be considered systematic.

## Conclusions

OQ_GAW_ and ClQ increase during and decrease immediately after water resistance therapy. However, the 30 min after WRT OQ_GAW_ rises could be interpreted as sign of vocal fatigue or vocal effort. Therefore, 10 min WRT with the tube 5 cm under water might provoke too much vocal loading for participants presenting with vocal fold mass legions suitable for treatment with phonomicrosurgery.
